# DNA barcoding of perennial fruit tree species of agronomic interest in the genus *Annona* (Annonaceae)

**DOI:** 10.3389/fpls.2015.00589

**Published:** 2015-07-30

**Authors:** Nerea Larranaga, José I. Hormaza

**Affiliations:** Instituto de Hortofruticultura Subtropical y Mediterránea La Mayora (IHSM-Universidad de Málaga, Consejo Superior de Investigaciones Científicas)Málaga, Spain

**Keywords:** *Annona*, DNA barcoding, matK, rbcL, species identification

## Abstract

The DNA barcode initiative aims to establish a universal protocol using short genetic sequences to discriminate among animal and plant species. Although many markers have been proposed to become the barcode of plants, the Consortium for the Barcode of Life (CBOL) Plant Working Group recommended using as a core the combination of two portions of plastid coding region, rbcL and matK. In this paper, specific markers based on matK sequences were developed for 7 closely related *Annona* species of agronomic interest (*Annona cherimola, A. reticulata, A. squamosa, A. muricata, A. macroprophyllata, A. glabra*, and *A. purpurea*) and the discrimination power of both rbcL and matK was tested using also sequences of the genus *Annona* available in the Barcode of Life Database (BOLD) data systems. The specific sequences developed allowed the discrimination among all those species tested. Moreover, the primers generated were validated in six additional species of the genus (*A. liebmanniana, A. longiflora, A. montana, A. senegalensis, A. emarginata* and *A. neosalicifolia*) and in an interspecific hybrid (*A. cherimola* x *A. squamosa*). The development of a fast, reliable and economic approach for species identification in these underutilized subtropical fruit crops in a very initial state of domestication is of great importance in order to optimize genetic resource management.

## Introduction

*Annona* L. is the type genus among the approximately 110 included in the Annonaceae (Chatrou et al., 2012), an angiosperm family within the Magnoliales in the Magnoliid clade (APG, 2009). Due to its phylogenetic situation among the early-divergent angiosperms, this family has been subject of a considerable interest from taxonomic and evolutionary points of view. Different molecular markers based on both chloroplast and nuclear sequences have been used to infer phylogenetic relationships among species of the family. Among chloroplast sequences, rbcL, matK, ndhF, trnL, trnT-L, trnL-F, trnS-G, atpB-rbcL, psbA-trnH, ycf1, rpl32-trnL, or ndhF-rpl32 have been used in one or several studies (Richardson et al., 2004; Chatrou et al., 2012; Thomas et al., 2012; Chaowasku et al., 2014). Among nuclear markers, microsatellite flanking regions were used by Chatrou et al. (2009). Additional molecular markers have been developed in the *Annona* genus mainly for fingerprinting and genetic diversity studies; isozymes (Ellstrand and Lee, 1987; Pascual et al., 1993; Perfectti and Pascual, 1998, 2004, 2005), randomly amplified polymorphic DNA (RAPDs) (Ronning et al., 1995), amplified fragment length polymorphism (AFLPs) (Rahman et al., 1998) and, more recently, microsatellites (Escribano et al., 2004, 2007, 2008a,b; Pereira et al., 2008; van Zonneveld et al., 2012).

Several species of the genus *Annona* produce edible fruits and have been cultivated and used as a food source by pre-Columbian cultures in Central and South America (Popenoe, 1989). Cultivation has continued to the present day and some of them now are incipient but prosperous crops in several developing countries with tropical and subtropical climates: cherimoya (*Annona cherimola* Mill.), sugar apple (*A. squamosa* L.), atemoya (*Annona* x *atemoya* Mabb., a hybrid between *A. cherimola* and *A. squamosa*), guanabana or soursop (*A. muricata* L.), custard apple (*A. reticulata* L.), ilama (*A. macroprophyllata* Donn. Sm.), pond-apple (*A. glabra* L.) or soncoya (*A. purpurea* Moc. & Sessé ex Dunal). All of them are native of the Neotropics and only *A. cherimola* is adapted to subtropical climates in higher elevations of Central and South America (from 1000 to 2500 m). Among those, cherimoya, sugar apple, guanabana, and atemoya show some commercial importance in various tropical and subtropical regions whereas the other species are generally used only locally with very limited or nonexistent production at a commercial scale. Production data are difficult to find for those species with perhaps the exception of cherimoya which value chains in South American countries have been recently studied (Vanhove and van Damme, 2013). The presence of interspecific hybrids and the lack of flowers and fruits during most of the year make rapid unequivocal identification of these species difficult in the field, hindering studies in different areas such as diversity evaluation and germplasm collection, conservation and management. Consequently a fast and reliable molecular method for identification of closely related species of *Annona* will be an important advance in those kinds of studies.

One of the best approaches to unequivocally identify species from leaf samples collected in the field is the use of DNA barcoding techniques. DNA barcoding aims to the adoption, in a great scale, of a few short standardized genome portions that allows a complete species identification and discrimination, especially in cases where morphological identification is difficult. In 2004 the international initiative Consortium for the Barcode of Life (CBOL) was founded for the development of a global method for the identification of plant and animal species. Six years later, the International Barcode of Life (iBOL) was activated for the maintenance of the barcode reference library BOLD (Barcode of Life Data systems) (Ratnasingham and Hebert, 2007; Bhargava and Sharma, 2013). Since Hebert et al. (2003) proposed the mitochondrial gene cytochrome c oxidase 1 (CO1) as the barcode for animal species, it has been widely used (Hebert et al., 2003; Nicolas et al., 2012). In the case of plants, the CBOL Plant Working Group recommended a two locus combination of the chloroplast ribulose-1, 5-bisphosphate carboxylase/oxygenase large subunit gene (*rbcL*) and maturase K gene (*matK*) as the core barcode (CBOL Plant Working Group, 2009). However, in some cases those two markers produce incomplete species resolution, especially in closely related taxa (Zhang et al., 2012) or recently diverged species (van Velzen et al., 2012). Consequently, the use of additional genome portions such as the internal transcribed spacer (*ITS*) and the second internal transcribed spacer (*ITS2*) from nuclear ribosomal DNA or the chloroplast *psbA*-*trnH* intergenic spacer have been proposed (Kress et al., 2005; Chen et al., 2010; China Plant BOL Group et al., 2011; Hollingsworth, 2011; Pang et al., 2012). In addition, the complete chloroplast genome is increasingly being used as super-barcode due to its species discrimination power, resolving some problems derived from the single or multiple loci barcode techniques (Li et al., 2015).

In order to have a fast and reliable method to discern between closely related *Annona* species with agronomic interest, in this work we developed species specific primers to unequivocally differentiate the seven most common agronomically interesting *Annona* species present in Central and South America (*Annona cherimola, A. reticulata, A. squamosa, A. muricata, A. macroprophyllata, A. glabra* and *A. purpurea*) based on newly sequenced data. The sequences were validated in 6 additional species of *Annona* and in an interspecific hybrid. The discrimination power of the most used plant barcode genes (rbcL and matK) was tested, using also additional *Annona* sequences available in the BOLD database and new sequences obtained were registered in the GenBank data base web page.

## Materials and methods

### Plant material

Leaves from three different genotypes of each of seven *Annona* species (*Annona cherimola, A. reticulata, A. squamosa, A. muricata, A. macroprophyllata, A. glabra*, and *A. purpurea*) were sampled in order to detect intraspecific variability. The materials were either collected from the wild or from an ex situ *Annona* germplasm collection maintained at the IHSM la Mayora in Málaga (Spain) at latitude 36°45′N, longitude 4°4′ W and altitude 35 m above sea level. Their geographic origin and codes are described in Table 1. All trees were previously identified in the field using morphological characters. Leaf samples from 6 additional *Annona* species (*A. liebmanniana* Baill.*, A. longiflora* S. Watson, *A. montana* Macfad., *A. senegalensis* Pers.*, A. emarginata* (Schltdl.) H. Rainer, and *A. neosalicifolia* H. Rainer.) and 5 atemoya hybrids (*A. cherimola* x *A. squamosa*), were used in order to test the validity of the primers generated (Table 1).

**Table 1:** **Information of the plant material used**.

					**GenBank accession number**	
**Species**	**Article code**	**GB code**	**Country of origin**	**Coordinates (Longitude/Latitude)**	**matK**	**rbcL**
*A. cherimola*	Che1	FDJ	Spain	−4.0438∕36.76006	KM068846	KM068867
*A. cherimola*	Che2	SP74	Peru	−4.0439∕36.7604	KM068847	KM068868
*A. cherimola*	Che3	Wild	Honduras	−88.17990∕14.31218	KM068848	KM068869
*A. reticulata*	Ret1	Wild	Honduras	−88.59383∕14.57977	KM068849	KM068870
*A. reticulata*	Ret2	Wild	Honduras	−87.18667∕14.56833	KM068850	KM068871
*A. reticulata*	Ret3	Wild	Guatemala	−90.00049∕14.91117	KM068851	KM068872
*A. squamosa*	Squ1	Aus2	Australia	−4.04158∕36.75799	KM068852	KM068873
*A. squamosa*	Squ2	Asquts	Unknown	−4.04116∕36.75443	KM068853	KM068874
*A. squamosa*	Squ3	Wild	Honduras	−87.64287∕14.56442	KM068854	KM068875
*A. muricata*	Mur1	Amur1	Unknown	−4.04146∕36.75797	KM068855	KM068876
*A. muricata*	Mur2	Wild	Honduras	−87.21413∕14.07677	KM068856	KM068877
*A. muricata*	Mur3	Wild	Guatemala	−90.88593∕14.10615	KM068857	KM068878
*A. macroprophyllata*	Mac1	Amacro3	Honduras	−4.04212∕36.76117	KM068858	KM068879
*A. macroprophyllata*	Mac2	Wild	Honduras	−88.93664∕14.55843	KM068859	KM068880
*A. macroprophyllata*	Mac3	Wild	Guatemala	−91.75712∕15.83533	KM068860	KM068881
*A. glabra*	Gla1	Agla2	Unknown	−4.04180∕36.75841	KM068861	KM068882
*A. glabra*	Gla2	Wild	Honduras	−88.86609∕15.17136	KM068862	KM068883
*A. glabra*	Gla3	Agla4	Unknown	−4.04206∕36.7611	KM068863	KM068884
*A. purpurea*	Pur1	Wild	Honduras	−87.65640∕14.60604	KM068864	KM068885
*A. purpurea*	Pur2	Wild	Honduras	−87.94751∕14.68543	KM068865	KM068886
*A. purpurea*	Pur3	Wild	Costa Rica	−84.95000∕10.78333	KM068866	KM068887
*A. liebmanniana*	Lie1	Wild	Honduras	−87.43655∕15.79678		
*A. longiflora*	Lon1	MA25S1	Mexico	−4.04224∕36.76108		
*A. montana*	Mon1	Amon1	Unknown	−4.04178∕36.75845		
*A. senegalensis*	Sen1	Asen1	Unknown	−4.04187∕36.7612		
*A. emarginata*	Ema1	Aema21	Paraguay	−4.04212∕36.76102		
*A. neosalicifolia*	Sal1	Aneo20	Paraguay	−4.04124∕36.75969		
Atemoya	Ate1	JT3	Breeding material	−4.04125∕36.75475		
Atemoya	Ate2	JT7	Breeding material	−4.04113∕36.7548		
Atemoya	Ate3	JT153	Breeding material	−4.04132∕36.75449		
Atemoya	Ate4	Ate Roja	Breeding material	−4.04147∕36.75802		
Atemoya	Ate5	19Joy	Breeding material	−4.04204∕36.75521		

*GB code refers to the code of the Germplasm Bank for the ex situ conserved material; “wild” stands for accessions not conserved ex situ*.

### DNA extraction, PCR amplification, visualization and sequencing

Plant DNA extraction was performed from 50 mg of young leaf tissue by a modified CTAB method (Viruel and Hormaza, 2004). Each PCR reaction contained 16 mM (NH_4_)_2_SO_4_, 67 mM Tris-ClH pH 8.8, 0.01% Tween-20, 3 mM MgCl_2_, 0.1 mM each dNTP, 0.3 μM each primer, 20 ng genomic DNA and 1 unit of BioTaq™ DNA polymerase (Bioline, London, UK) in a final volume of 15 μl. For sequencing the final volume was increased to 35 μl maintaining the concentration of the different ingredients. Universal primers and PCR protocols used to amplify rbcL and matK genes were those proposed by the Plant Working Group of the Consortium for the Barcode of Life (www.barcoding.si.edu/plant_working_group.html). Primers developed in this work are described in Table 2. PCRs were carried out in an I-cycler (Bio-Rad Laboratories, Hercules, CA, USA) thermocycler using the following temperature profile: an initial step of 1 min at 94°C, 35 cycles of 30 s at 94°C, 30 s at 60–69°C (Table 2), and 1 min at 72°C, and a final step of 5 min at 72°C. Amplicons were visualized in 1 or 3% agarose gels stained with Gel Red (1X).

**Table 2:** **Details of the primers designed for identification of species of ***Annona*** including sequence, PCR melting and annealing temperatures, expected PCR product size and target species**.

**Name**	**Sense**	**Sequence 5′-3′**	**Melting temp. (° C)**	**Annealing temp. (°C)**	**Expected size (bp)**	**Specificity**
AChF1	F	GTATATGAATGTGAATCGGTATTC	58.3	65	396	*Annona cherimola matK*
AChR1	R	TTGACTCCTTACTGCGGAAT	61.7			
AChReF1	F	GCTTCGGAATGATTTTCC	60.1	65	364	*Annona reticulata matK*
AReR1	R	CGCCTTAGCCAACGATT	61.9			
ASqF1	F	CCATTTCCGTTTGTTCAAAC	62.2	69	315	*Annona squamosa matK*
ASqR1	R	GGTAAGATTTCCATTTCTTCATC	59.8			
AMuF1	F	CATTTACGATCAACATCCTTTA	58.6	65	332	*Annona muricata matK*
AMuR1	R	GAAGAATTTTGGCGTACACTTA	60.2			
AMaF1	F	ATACAAGATGCTCCCTCTTTG	60.2	69	644	*Annona macroprophyllata matK*
AMaR1	R	TTAGCCAATGATCCAATCATT	61.2			
AGlF1	F	CGATCAACATCCTTTGGG	62.1	69	476	*Annona glabra matK*
AGlR1	R	GCCGGCTTACTAATAGGGTT	61.3			
APuF1	F	TTCTTGTTCCTATATAATATTCATA	53.2	61	619	*Annona purpurea matK*
APuR1	R	GAGAAAGATTTCTGTATATGCGT	58.5			
1R_kim	F	ACCCAGTCCATCTGGAAATCTTGGTTC		52		*Universal matK*
3F_kim	R	CGTACAGTACTTTTGTGTTTACGAG				
rbcLa_F	F	ATGTCACCACAAACAGAGACTAAAGC		55–54		*Universal rbcL*
rbcLa_R	R	GTAAAATCAAGTCCACCRCG				

Two multiplex PCR methods were developed for 6 of the 7 specific markers, depending on their annealing temperature. At 65°C, primers AChF1, AChR1, AChReF1, AReR1, AMuF1, AMuR1 were placed in the same master PCR mix, only decreasing the concentration of AChF1 and AChR1 from 0.3 to 0.07 μM. At 69°C, primers ASquF1, ASquR1, AMaF1, AMaR1, AGlaF1, AGlaR1 were also used together increasing every concentration from 0.3 to 0.4 μM.

Prior to sequencing, PCR solutions were purified with NucleoSpin® Extract II (Macherey-Nagel). Sequencing was done by the fluorescent dye terminator sequencing method (Macrogen).

### Barcode sequence quality and species discrimination

Amplified matK and rbcL of the 21 different *Annona* DNA samples belonging to 7 species were sequenced in the forward direction. Chromatograms were analyzed by Chromas Lite version 2.1 (2012). The criteria used to establish an acceptable sequence were as follows (modified from Hanner, 2009): (i) ends were trimmed to minimize low quality base calls on each end; all bases were deleted until there were at least contiguous 10 pair bases with a PHRED score >20 (ii) bases with PHRED values less than 20 were recorded as N; (iii) sequences with more than 40% N base calls were deleted.

**Figure 1:**
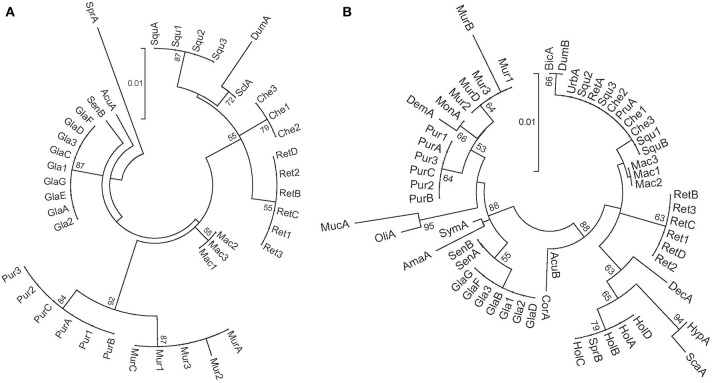
**Neighbor joining trees of pairwise K2P substitution rates using 41 matK sequences (A) and 57 rbcL sequences (B)**. Taxon codes are indicated in Table 1 and Supporting information 1. Bootstrap values of 50% and above are shown on the clusters. The scale bar represents the substitution rate per site.

To assess the discriminatory power of both genes at the specific level, the 42 sequences obtained, 21 of matK and 21 of rbcL, were aligned with MUSCLE implemented in the software MEGA5 version 5.05 (Tamura et al., 2011) using the default options. Pairwise sequence distances were calculated using the Kimura-2-parameter (K2P) that takes into account transitions and transversions, with the same software (Aubriot et al., 2013). For each species, when the maximum intraspecific distance was lower than the minimum interspecific distance, discrimination was considered successful (CBOL Plant Working Group, 2009). In addition, a tree based identification approach was used. For that, all sequences of the genus *Annona* available in BOLD for each of the two genes analyzed were downloaded (access 08/2012) (Ratnasingham and Hebert, 2007); in total 20 sequences of matK belonging to 10 different species and 36 sequences of rbcL belonging to 24 different species were available (Supporting Information 1). Two Neighbor Joining (NJ) trees of K2P distances were constructed using the same program and running 1000 bootstrap replicates (Figure 1).

### Design of species-specific markers

In order to design PCR-specific primers, newly sequenced alignments for matK and rbcL *Annona* DNA samples and the sequences downloaded from BOLD were used. Inter-species polymorphisms were studied manually using GENEDOC version 2.7. Intraspecific variations were not considered. The gene matK was selected for the development of 7 *Annona* specific markers. Pairs of specific primers were designed locating the 3′ end of each primer in specific shared polymorphic regions compared to the sequences that belonged to all the other species. Primers were designed to amplify targets of different length so PCR output could be determined by its position in a gel. To calculate melting temperatures and avoid as much as possible interaction among primers (also within different markers) we used the Multiple Primer Analyzer available at http://www.thermoscientificbio.com/webtools/multipleprimer (access 02/2013). Primers designed are described in Table 2 and were validated in 6 additional *Annona* species through PCR amplification of DNA and visualization of the amplification fragments in agarose gels.

## Results

### Barcode sequences quality and species discrimination

All of the 42 sequences obtained fulfilled the established criteria to consider the sequencing reactions successful. Sequence sizes after the trimming process varied from 752 to 820 bp in the case of matK and from 470 to 528 in the case of rbcL. The number of bases with a PHRED score below 20, so, therefore, substituted by N, was between 0 and 7, with the exception of sample Mur2 amplified with matK primers which had 61 bad quality bp (less of 40% of the sequence). All sequences were uploaded to the GenBank database and their accession numbers are indicated in Table 1.

Intraspecific sequence variability was only found in *A. muricata*. The maximum intraspecific distance was then compared to the minimum interspecific distance; in almost all cases the former was smaller than the latter (Table 3). Nucleotide sequence of the barcode gene matK could discriminate all 7 species, while rbcL could not distinguish between *A. cherimola* and *A. squamosa* samples.

**Table 3:** **Intra- and interspecific 2KP distances among the new sequences obtained**.

	**matK**				**rbcL**			
**Species (codes)**	**Intraspecific distances**		**Interspecific distances**		**Intraspecific distances**		**Interspecific distances**	
	**Min**	**Max**	**Min**	**Max**	**Min**	**Max**	**Min**	**Max**
*A. cherimola* (Che1, Che2, Che3)	0.000	0.000	0.008	0.023	0.000	0.000	0.000	0.013
*A. reticulata* (Ret1, Ret2, Ret3)	0.000	0.000	0.009	0.024	0.000	0.000	0.002	0.015
*A. squamosa* (Squ1, Squ2, Squ3)	0.000	0.000	0.008	0.021	0.000	0.000	0.000	0.013
*A. muricata* (Mur1, Mur2, Mur3)	0.000	0.001	0.009	0.023	0.000	0.000	0.004	0.015
*A. macroprophyllata* (Mac1, Mac2, Mac3)	0.000	0.000	0.011	0.023	0.000	0.000	0.002	0.023
*A. glabra* (Gla1, Gla2, Gla3)	0.000	0.000	0.014	0.018	0.000	0.000	0.009	0.018
*A. purpurea* (Pur1, Pur2, Pur3)	0.000	0.000	0.009	0.024	0.000	0.000	0.009	0.024

The NJ trees obtained are shown in Figure 1. In the matK tree, new sequences from the 7 tested species grouped in different clusters. Besides, where applicable, sequences from the same species (newly obtained in this work and downloaded from BOLD) grouped together. In the case of rbcL, all the 21 new sequences clustered by species except *A. cherimola* and *A. squamosa*, that were grouped together. Furthermore, sequences from the same species downloaded from BOLD and the newly obtained sequences grouped together in the same cluster. Nevertheless, some species were mixed; in the cluster of *A. cherimola* and *A. squamosa* samples, three additional species (*A. urbaniana, A. reticulata* and *A. pruinosa*) downloaded from BOLD were present. *A. holosericea* and *A. spraguei* BOLD samples appeared also in the same cluster.

### Specific markers

Seven markers were developed for 7 different *Annona* species. Details on the primer features and amplification conditions are summarized in Table 2. Primer specificity was verified using 13 samples of different *Annona* species (Supporting Information 2). For each species, just one band appeared when the specific markers developed were used.

In order to optimize routine species identification two multiplex PCR methods were developed. One for *A. squamosa, A. macroprophyllata* and *A. glabra* markers (all with 69°C annealing temperature), whose expected size amplicons are 315, 644, and 476 bp respectively. The other, for *A. cherimola, A. muricata* and *A. reticulata* markers (all with 65°C annealing temperature), whose expected size amplicons are 396, 332, and 364 bp respectively. In addition, AChReF1 hybridizes with *A. cherimola* sequences so, when added to the same reaction than the AChR1 markers, a new band of 271 bp appeared in the *A. cherimola* samples (Supporting Information 3).

These markers could also be used to study interspecific hybrids. Five atemoya samples, a hybrid of *A. cherimola* and *A. squamosa*, were analyzed with both loci. Four of the hybrid atemoyas where known to have *A. cherimola* as maternal parent and, as expected, those 4 samples only showed amplification bands when amplified with the *A. cherimola* specific primers (Supporting Information 4). The fifth sample, for which the direction of the cross was unknown, also showed this band when amplified with the *A. cherimola* specific marker, indicating *A. squamosa* as the pollen donor.

## Discussion

In this work we tested the two chloroplast loci, rbcL and matK, proposed for the barcode of plants by the CBOL (CBOL Plant Working Group, 2009), for differentiation of species of agronomic interest in the genus *Annona*. Sequences of other species of the genus present in the BOLD database were also included in the analysis. The results obtained by distance analysis methods with 21 genotypes belonging to 7 agronomical important *Annona* species showed almost no intraspecific variation, which could be due to the limited number of samples analyzed. One criticism of this kind of analyses is that often a very few individuals per species are studied or that incomplete geographic sampling is performed resulting in possible poor representation of the intraspecific variation or interspecific divergence, given rise to possible misleading results regarding barcoding gap and overlap between intra- and interspecific variation (Meyer and Paulay, 2005). Although for some species the samples used here were obtained from different geographical areas, in other species the samples used just cover a small part of their distribution range; consequently, additional intraspecific variation could be present if samples from other regions were included in the analysis. Locus rbcL could distinguish 5 of the 7 species, showing overlap between intra- and interspecific variation, while locus matK could distinguish all of them, since intraspecific distances were smaller than interspecific values, showing a barcode gap with the number of species studied. Both rbcL and matK have been recommended as core plant barcodes by CBOL (CBOL Plant Working Group, 2009) although, as shown in this work, rbcL usually displays a lower interspecific variation (Kress et al., 2005). Moreover, the universality of these two markers has been questioned in some particular taxa (Chase et al., 2007; Roy et al., 2010).

This initial analysis was broadened with comparison with additional sequences from other species of the genus *Annona* available in the BOLD database using NJ trees. Tree-based methods are also used for the identification of query samples when comparing its barcode sequences to a data set and the clustering position analyzed (van Velzen et al., 2012). Results, again, show a good performance of both genes, particularly of matK, since sequences from different individuals of the same species were grouped together in different clusters of the NJ tree. The mixed presence of some species in the NJ trees could be explained by this lower identification power of rbcL (when this number of different species is used) or by errors in species identification prior to uploading the sequences to the BOLD database. A multilocus analysis was not carried out since matK could discriminate all the species studied.

Newly obtained and BOLD matK sequences alignment was also used for the manual finding of specific polymorphic nucleotides. These were used for the development of 7 specific markers, relying on the idea of diagnostic positions. In fact, character-based diagnosis methods are the other big group used in traditional barcode techniques. They count just on relevant diagnostic positions of the sequence that would support a particular classification (DeSalle et al., 2005).

In the case of *Annona* species, the development of fast, reliable and economic tools, such as specific markers, to identify these promising subtropical resources in a very initial state of domestication is of great importance in order to optimize genetic resource management. In addition, since these markers are chloroplast based genes, their presence or absence in interspecific hybrids could serve to discern hybridization processes in these species; some of them are known to hybridize easily (such as *A. cherimola* and *A. squamosa*) although some authors (Zill and Mahdeem, 1998; George et al., 2002) have reported other hybrids in the genus (such as *A. cherimola* x *A. reticulata* or *A. squamosa* x *A. diversifolia*) at least as a result of artificial pollination. The markers described in this work are appropriate for the discrimination of the most important species of the genus from an agronomic point of view although, since the genus *Annona* includes about 160 species (Chatrou et al., 2012), additional work will be needed to develop appropriate identification tools for all the species of the genus.

## Author contributions

JH and NL conceived, designed the study and wrote the paper. NL performed the molecular experiments and analyzed the data.

### Conflict of interest statement

The authors declare that the research was conducted in the absence of any commercial or financial relationships that could be construed as a potential conflict of interest.
