# Supplementary material for: DNA barcoding of perennial fruit tree species of agronomic interest in the genus Annona (Annonaceae)
Source: Front Plant Sci. 2015 Jul 30;6:589. doi: 10.3389/fpls.2015.00589 (PMC4519677; doi:10.3389/fpls.2015.00589)
Supplement: Supplementary file 1 [file Table1.DOCX]

**Table S1.** MatK and rbcL BOLD sequences downloaded from http://www.boldsystems.org/

| **BOLD Code** | **Code** | **Species** | **rbcL (bp)** | **matK (bp)** | **Collected in** | |
| --- | --- | --- | --- | --- | --- | --- |
| GBVG4966-11 | AcuA | *A. acuminata* |  | 566 | Panama |  |
| GBVG4967-11 | AcuB | *A. acuminata* | 553 |  | Panama |  |
| GBVG4968-11 | AmaA | *A. amazonica* | 1317 |  | Bolivia |  |
| GBVG4969-11 | BicA | *A. bicolor* | 1369 |  | Mexico |  |
| GBVG4970-11 | CorA | *A. cornifolia* | 684 |  | Bolivia |  |
| GBVG4971-11 | DecA | *A. deceptrix* | 1427 |  | Ecuador |  |
| GBVG4972-11 | DemA | *A. deminuta* | 1291 |  | Peru |  |
| GBVG4973-11 | DumA | *A.dumetorum* |  | 978 | - |  |
| GBVG4974-11 | DumB | *A.dumetorum* | 1368 |  | Dominican Republic |  |
| GBVG4975-11 | GlaA | *A. glabra* |  | 831 | United States |  |
| GBVG4976-11 | GlaB | *A. glabra* | 1427 |  | United States |  |
| GBVG4977-11 | GlaC | *A. glabra* |  | 1524 | - |  |
| GBVG4978-11 | HolA | *A. holosericea* | 1369 |  | Honduras |  |
| GBVG4979-11 | HypA | *A. hypoglauca* | 1369 |  | Bolivia |  |
| GBVG4980-11 | MonA | *A. montana* | 1369 |  | - |  |
| GBVG4981-11 | MurA | *A. muricata* |  | 1524 | - |  |
| GBVG4982-11 | MurB | *A. muricata* | 1400 |  | - |  |
| GBVG4983-11 | MurC | *A. muricata* |  | 831 | - |  |
| GBVG4984-11 | MurD | *A. muricata* | 1421 |  | - |  |
| GBVG4985-11 | OliA | *A. oligocarpa* | 1269 |  | Ecuador |  |
| GBVG4986-11 | PruA | *A. pruinosa* | 1369 |  | Costa Rica |  |
| GBVG4987-11 | RetA | *A. reticulata* | 1369 |  | Bolivia |  |
| GBVG4988-11 | ScaA | *A. scandens* | 676 |  | Bolivia |  |
| GBVG4989-11 | SclA | *A. sclerophylla* |  | 1524 | - |  |
| GBVG4990-11 | SenA | *A. senegalensis* | 1426 |  | - |  |
| GBVG4991-11 | SprA | *A. spraguei* |  | 783 | Panama |  |
| GBVG4992-11 | SprB | *A. spraguei* | 550 |  | Panama |  |
| GBVG4993-11 | SquA | *A. squamosa* |  | 842 | - |  |
| GBVG4994-11 | SquB | *A. squamosa* | 1369 |  | Netherlands Antilles |  |
| GBVG4995-11 | SymA | *A. symphyocarpa* | 1343 |  | Guyana |  |
| GBVG4996-11 | UrbA | *A. urbaniana* | 1369 |  | Dominican Republic |  |
| INB080-12 | MucA | *A. mucosa* | 552 |  | Costa Rica |  |
| KNPA853-09 | SenB | *A. senegalensis* | 555 | 1056 | South Africa |  |
| MHPAC1050-11 | HolB | *A. holosericea* | 552 |  | Costa Rica |  |
| MHPAC1051-11 | HolC | *A. holosericea* | 552 |  | Costa Rica |  |
| MHPAC1052-11 | HolD | *A. holosericea* | 552 |  | Costa Rica |  |
| MHPAC370-08 | RetB | *A. reticulata* | 553 | 1006 | Costa Rica |  |
| MHPAC371-08 | RetC | *A. reticulata* | 552 | 1006 | Costa Rica |  |
| MHPAC378-08 | RetD | *A. reticulata* | 552 | 975 | Costa Rica |  |
| MHPAC397-08 | PurA | *A. purpurea* | 551 | 1006 | Costa Rica |  |
| MHPAC398-08 | PurB | *A. purpurea* | 552 | 1006 | Costa Rica |  |
| MHPAC399-08 | PurC | *A. purpurea* | 552 | 1006 | Costa Rica |  |
| MHPAD3022-10 | GlaD | *A. glabra* | 552 | 1006 | Costa Rica |  |
| MHPAD3023-10 | GlaE | *A. glabra* |  | 1006 | Costa Rica |  |
| MHPAD3024-10 | GlaF | *A. glabra* | 552 | 995 | Costa Rica |  |
| MHPAD3025-10 | GlaG | *A. glabra* | 552 | 1006 | Costa Rica |  |
