# Supplementary material for: DNA barcoding of perennial fruit tree species of agronomic interest in the genus Annona (Annonaceae)
Source: Front Plant Sci. 2015 Jul 30;6:589. doi: 10.3389/fpls.2015.00589 (PMC4519677; doi:10.3389/fpls.2015.00589)

Fig. S2. Electrophoresis in 3% agarose gel of 2 multiplex PCR. Taxon codes are indicated in Table 1. W: water. 1) Multiplex PCR with *A. cherimola*, *A. reticulata* and *A. muricata* primers. 2) Multiplex PCR with *A. squamosa*, *A. macroprophyllata* and *A. glabra* primers. Hiperladder 1Kb of Bioline was used as size marker.

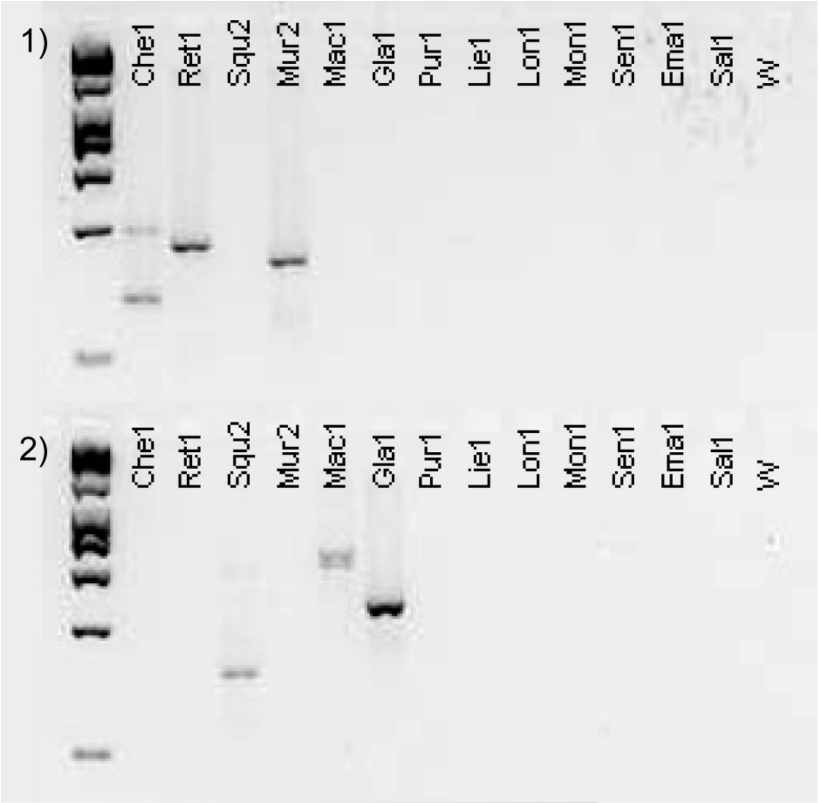

Supplement: Supplementary file 3 [file Image2.PDF]
