# Supplementary material for: DNA barcoding of perennial fruit tree species of agronomic interest in the genus Annona (Annonaceae)
Source: Front Plant Sci. 2015 Jul 30;6:589. doi: 10.3389/fpls.2015.00589 (PMC4519677; doi:10.3389/fpls.2015.00589)

Fig. S3. Electrophoresis in 1% agarose gel of *A. cherimola* and *A. squamosa* primers with atemoya DNA samples. Taxon codes are indicated in Table 1. W: water. Hiperladder 1Kb of Bioline was used as size marker.

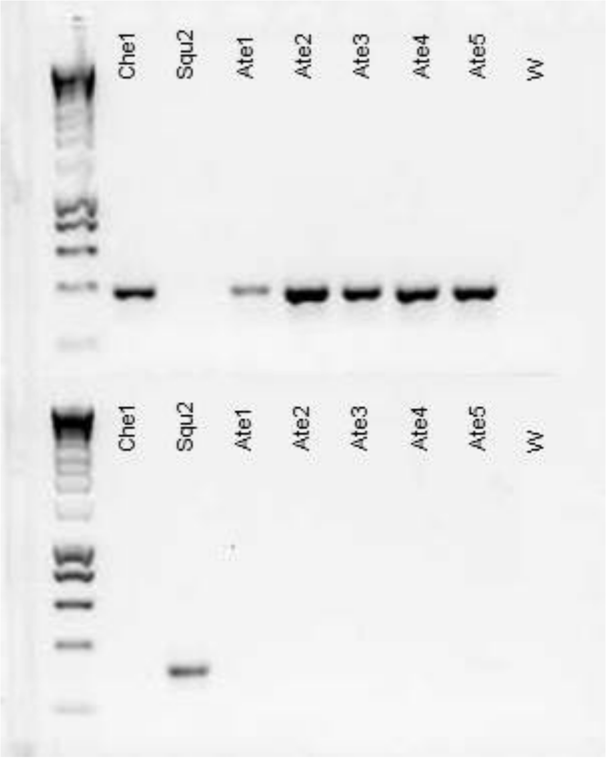

Supplement: Supplementary file 4 [file Image3.PDF]
