# Supplementary material for: DNA barcoding of perennial fruit tree species of agronomic interest in the genus Annona (Annonaceae)
Source: Front Plant Sci. 2015 Jul 30;6:589. doi: 10.3389/fpls.2015.00589 (PMC4519677; doi:10.3389/fpls.2015.00589)

Fig. S1. Electrophoresis in 1% agarose gels of specific PCR reactions using the primers developed in this work and 13 DNA samples from different species in the *Annonaceae*. Taxon codes are indicated in Table 1. W: water. 1) PCR with universal matK primers, 2) PCR with *A. cherimola* primers, 3) PCR with *A. reticulata* primers, 4) PCR with *A. squamosa* primers, 5) PCR with *A. muricata* primers, 6) PCR with *A. macroprophyllata* primers, 7) PCR with *A. glabra* primers, 8) PCR with *A. purpurea* primers. Hiperladder 1Kb of Bioline was used as size marker.

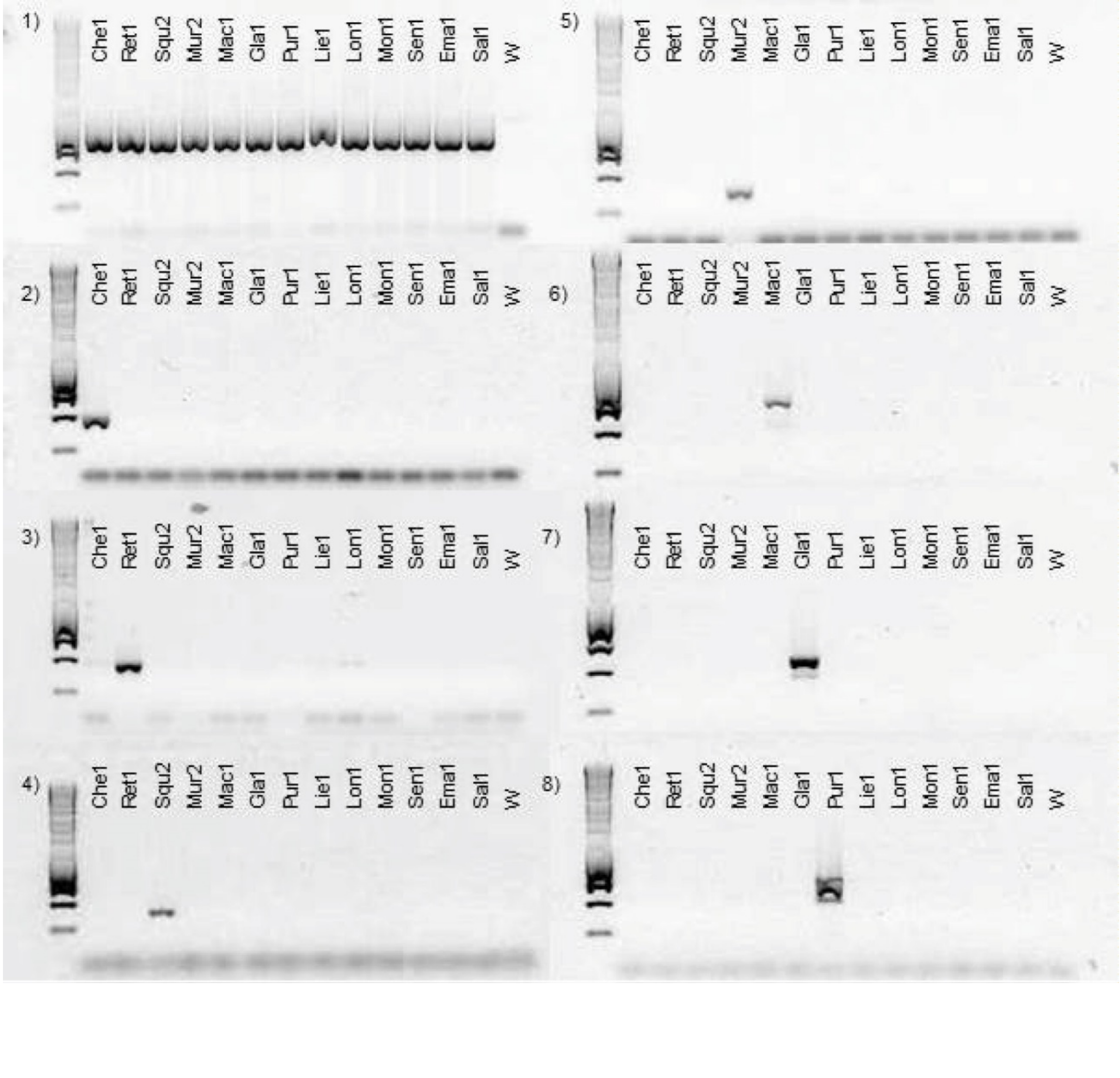

Supplement: Supplementary file 2 [file Image1.PDF]
